# Evaluation of primary care midwifery in the Netherlands: design and rationale of a dynamic cohort study (DELIVER)

**DOI:** 10.1186/1472-6963-12-69

**Published:** 2012-03-20

**Authors:** Judith Manniën, Trudy Klomp, Therese Wiegers, Monique Pereboom, Johannes Brug, Ank de Jonge, Margreeth van der Meijde, Eileen Hutton, Francois Schellevis, Evelien Spelten

**Affiliations:** 1 Department of Midwifery Science, AVAG and the EMGO Institute for Health and Care Research, VU University Medical Center, Amsterdam, the Netherlands; 2 Netherlands Institute for Health Services Research (NIVEL), Utrecht, the Netherlands; 3 EMGO Institute for Health and Care Research, VU University Medical Center, Amsterdam, the Netherlands; 4 Institute for Training and Education, VU University Medical Center, Amsterdam, the Netherlands; 5 Faculty of Health Sciences, McMaster University, Hamilton, Canada; 6 Department of General Practice/EMGO Institute for Health and Care Research, VU University Medical Center, Amsterdam, the Netherlands

## Abstract

**Background:**

In the Netherlands, midwives are autonomous medical practitioners and 78% of pregnant women start their maternity care with a primary care midwife. Scientific research to support evidence-based practice in primary care midwifery in the Netherlands has been sparse. This paper describes the research design and methodology of the multicenter multidisciplinary prospective DELIVER study which is the first large-scale study evaluating the quality and provision of primary midwifery care.

**Methods/Design:**

Between September 2009 and April 2011, data were collected from clients and their partners, midwives and other healthcare professionals across the Netherlands. Clients from twenty midwifery practices received up to three questionnaires to assess the expectations and experiences of clients (e.g. quality of care, prenatal screening, emotions, health, and lifestyle). These client data were linked to data from the Netherlands Perinatal Register and electronic client records kept by midwives. Midwives and practice assistants from the twenty participating practices recorded work-related activities in a diary for one week, to assess workload. Besides, the midwives were asked to complete a questionnaire, to gain insight into collaboration of midwives with other care providers, their tasks and attitude towards their job, and the quality of the care they provide. Another questionnaire was sent to all Dutch midwifery practices which reveals information regarding the organisation of midwifery practices, provision of preconception care, collaboration with other care providers, and provision of care to ethnic minorities. Data at client, midwife and practice level can be linked. Additionally, partners of pregnant women and other care providers were asked about their expectations and experiences regarding the care delivered by midwives and in six practices client consults were videotaped to objectively assess daily practice.

**Discussion:**

In total, 7685 clients completed at least one questionnaire, 136 midwives and assistants completed a diary with work-related activities (response 100%), 99 midwives completed a questionnaire (92%), and 319 practices across the country completed a questionnaire (61%), 30 partners of clients participated in focus groups, 21 other care providers were interviewed and 305 consults at six midwifery practices were videotaped.

The multicenter DELIVER study provides an extensive database with national representative data on the quality of primary care midwifery in the Netherlands. This study will support evidence-based practice in primary care midwifery in the Netherlands and contribute to a better understanding of the maternity care system.

## Background

In the Netherlands midwives are autonomous medical practitioners, qualified to provide full maternity care on their own accountability to all women whose pregnancy and childbirth are uncomplicated including prenatal, intrapartum and postnatal care to mother and child [1]. The first appointment at the midwifery practice usually takes place around the 8^th ^week of gestation. Because of the frequent (on average 13) contacts throughout pregnancy and because of their expertise, midwives are considered to be important reliable providers of pregnancy-related health education and advice for pregnant women.

In the Netherlands about 175,000 births occur annually. In 2009 there were 2444 registered and practising midwives (one per 1630 women within the fertile age range), of which 77% worked in a primary care setting, in just over 500 midwifery practices [2]. The vast majority of pregnant women (78%) start their maternity care in a primary care setting, 44% start labour in primary care, and eventually 33% of women give birth under supervision of a primary care midwife [3]. In order to be allowed to practice midwifery, midwives in the Netherlands are educated in a four year Bachelor level program in one of the four midwifery colleges in the Netherlands. Additionally, midwives can choose to follow a midwifery Master program.

Up to now, scientific research to support evidence-based practice in primary care midwifery in the Netherlands has been sparse. It is essential to accomplish more research that evaluates the maternity care system and practice, in order to develop a better understanding of the maternity system and to provide scientific knowledge for improvement.

Therefore, the Academy of Midwifery Amsterdam-Groningen (AVAG), the Netherlands Institute for Health Services Research (NIVEL), and the EMGO Institute for Health and Care Research of VU University Medical Centre initiated the national DELIVER-study. DELIVER is the Dutch acronym for data primary care midwifery (Data EersteLIjns VERloskunde).

The DELIVER study aims to gain insight into the quality, organisation and accessibility of midwifery care in the Netherlands. Results of the Deliver study should further improve midwifery care in the Netherlands and contribute to evidence-based practice. This paper describes the research design and methodology of this multicenter multidisciplinary prospective study.

### Research questions

The DELIVER study is primarily a descriptive study with the following research questions:

- How is primary care midwifery organised in the Netherlands?

- What is the accessibility of primary care midwifery in the Netherlands?

- What is the quality of primary care midwifery in the Netherlands?

Regarding the organisation of care, the DELIVER study aims to provide evidence about the referring system ('gate-keeper function' of midwives), role and responsibilities of midwives, collaboration with other care providers (e.g. continuity of care), and time expenditure of midwives. Regarding the accessibility of midwifery care, the study will assess the uptake of care (e.g. number of appointments, number of ultrasound scans, postnatal maternity care), number of ethnic minority women and undocumented women under care of a midwife, and accessibility of the practice (e.g. appointment times). The quality of primary care midwifery in the Netherlands from the preconception to postnatal period will be assessed by describing communication and provision of health information (e.g. information on prenatal screening, lifestyle, pain management, place of birth, labour positions), adherence to standards and guidelines, training and education of (student) midwives, experiences and satisfaction of clients (e.g. confidence in their midwife), and pregnancy outcomes. Additionally, data on midwives' attitudes towards their job and emotions, feelings, health and lifestyle of clients were collected to enable exploration of a range of secondary research questions.

## Methods/Design

### Study design

The DELIVER study was designed as a multicenter prospective dynamic cohort study to evaluate primary care midwifery in the Netherlands with the main focus on quality, organisation and accessibility of care. The maternity care system was assessed from the perspective of the clients as well as from the perspective of the midwives and other involved care providers. The dynamic cohort consisted of clients who completed up to three questionnaires between their first appointment in the midwifery practice and six weeks postpartum within an observation period of one calendar year. Of these clients, data were also obtained from the national Netherlands Perinatal Registry and from electronic client records kept by midwives. Data on midwifery practice were assembled by questionnaires, by recording work-related activities during one week, and by video-recordings of intake consults with clients. In addition, focus groups with client's partners evaluated their expectations, needs and experiences regarding midwifery care. Finally, interviews were held with other maternity care providers to gain insight into their experience regarding collaboration with midwives. The learning experiences of two National Surveys of General Practice, which were conducted by the NIVEL institute, were used to develop the design of the DELIVER study [4].

### Recruitment and enrolment of study participants

#### Recruitment and participation of midwifery practices

Midwives and their clients were recruited from twenty midwifery practices. Purposive sampling was used to select practices, using three stratification criteria: region (north, east, south, west), level of urbanisation (urban or rural area), and practice type (dual or group practice). Twenty of the 519 primary care practices in the Netherlands were approached and invited to participate in this study. The approached practices received a brochure with information on the study and were visited by two members of the DELIVER research team who explained the study in further detail. If a practice declined participation, a replacement was found taking region, urbanisation and practice type into account. Ultimately, fourteen practices declined participation, mostly because of time constraints. Each participating practice signed a contract through which they gave consent to cooperate in all parts of the study, including related studies by PhD students. The twenty participating practices comprised 108 midwives and about 8200 clients per year. Midwives were instructed to provide usual care to all their clients irrespective of their participation and to refer clients with questions about the study to the research team.

Of the twenty DELIVER practices, six gave permission to videotape intake consults with clients. Prior to taping, each client was asked for consent to videotape the consult.

#### Recruitment of clients

The client recruitment period at each midwifery practice was twelve months. Three practices started including patients in September 2009, two started in October 2009, thirteen in November 2009 and two in December 2009. In the first month at each practice, all clients in care were invited to participate, irrespective of their gestational age or whether they recently (maximum 6 weeks before) gave birth. The following months only new clients were invited to participate. Clients were eligible to participate if they were able to understand Dutch, English, Turkish or Arabic. The midwives were instructed to inform all eligible clients individually about the study and invite them to participate. The midwives gave all women who were interested a brochure about the study with a link to the website of the study [5] where they could find additional information about the study.

To improve the overall response, a reminder was sent to all non-responders. In addition, five research assistants were enrolled (student midwives) to call all clients who did not complete the questionnaire within one week and invite them once more to participate.

#### Recruitment of partners of clients

Partners of pregnant women were recruited in two midwifery practices in November and December 2010. During a consult, midwives informed the partners about the study and asked them whether they were interested. If the partners did not accompany their pregnant partner to a consult with the midwife, the midwife asked the woman whether she thought her partner might be interested in the study. Each practice sent a list of clients who were at least 28 weeks pregnant and whose partners were possibly interested in the study to a research bureau (Intomart GfK). The research bureau first sent a letter to the partners with information about the study and then phoned them to invite them to participate. One focus group was organised per practice for partners of women expecting their first child, and one for partners of women who already had at least one child. The four focus groups were undertaken and analysed by Intomart GfK.

#### Recruitment of other maternity care providers

Seven categories of maternity care providers were included: clinical midwives, gynaecologists, general practitioners, maternity care assistants, paediatricians, ambulance personnel, and Obstetrics&Gynaecology (O&G) nurses. Each of the twenty DELIVER practices provided information on three of their contacts per category. The research bureau Intomart GfK executed and analysed in total 21 telephone interviews (March and April 2011): four with general practitioners, two with ambulance personnel, and three for each other category. Care providers were selected randomly, stratified by urban and rural practices.

### Measurement tools

#### Measurement tools administered among midwives

**Figure 1:**
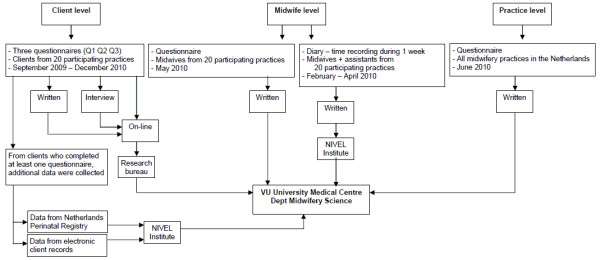
**Data flow of the questionnaires and diaries**.

All midwives and practice assistants of the twenty participating practices were requested to keep a diary for one week sometime between February and April 2010 (Figure 1). The goal of this diary was to get detailed insight into the real time expenditure of midwives for different responsibilities. Within each practice, all midwives and practice assistants were required to complete the diaries in the same week, choosing a week without public holidays. In the diary they used time sheets to record all work-related activities 24 hours per day for 7 days, using a pre-structured format. This pre-structured diary had been successfully used before by the NIVEL Institute in studies evaluating time expenditure of Dutch midwives [6]. The activities were categorized as midwifery clinic (e.g. consultation during pregnancy, ultrasound scan, or 6 weeks postnatal consultation), being on call, intrapartum care, postnatal care visits, hospital visits, administrative tasks, or meetings.

In addition, all midwives from the twenty participating practices were asked to complete a written questionnaire in May 2010 (Figure 1). The aim of this questionnaire was to gain insight into collaboration of midwives with other care providers, their attitude towards their job, and their adherence to standards and guidelines (Table 1). Another questionnaire was sent to all midwifery practices in the Netherlands in June 2010 (Figure 1). The questionnaires provide description of the size and organisation of midwifery practices, provision of preconception care, collaboration with other care providers, education of students, and provision of care to ethnic minority women and undocumented women (Table 1). Many questions from the midwife questionnaire and the practice questionnaire were derived from earlier studies [4,7].

**Table 1:** Content information of the questionnaires

Subjects	Moment	Number of items	Issues	Examples of questions
Clients	September 2009 to December 2010	Q0*: 32 Q1: 51 Q2: 73 Q3: 100	a. quality of midwifery care b. ultrasound scans and prenatal screening c. (preparation for) childbirth d. emotions and feelings e. health f. lifestyle g. questions specifically for clients from ethnic minority groups	a. Rate experiences with care provided by midwives/maternity care assistants (0 = worst possible care to 10 = best possible care; 10 steps) b. Did you choose to undergo one or more ultrasound scans, and with hindsight, would you have chosen differently? c. How much pain did you expect/experience? (0 = no pain to 10 = the worst imaginable pain; 20 steps) Have you drawn up a birth plan outlining your preferences and expectations during labour and delivery? d. Were you happy/afraid/worried during pregnancy? Did you feel tense/important/confident during labour? e. In general, how would you rate your current state of health? Do you suffer from any chronic illnesses, disorders or disabilities? f. Did you smoke, drink alcohol, use drugs during pregnancy? Do you take folic acid, vitamin B12, vitamin D? g. Are you planning to consult or have you consulted a midwife and/or gynaecologist in your or your parents' country of birth? Was your midwife and/or maternity care assistant sympathetic to ethnic differences in customs surrounding the birth?

Midwives from 20 participating practices	May 2010	115	a. collaboration with other health care providers b. tasks c. attitudes towards job d. quality of care e. general information	a. On average, how often do you have contact with a general practitioner, secondary care midwife, obstetrician, paediatrician, or maternity care assistant; for what reason do you have contact; who usually takes initiative for that; are you satisfied with the collaboration? b. How often do you provide psychosocial care to clients? How often do you discuss lifestyle (eating, drinking, smoking) with clients? During the last 6 months, how much time did you spend on meetings/organised information activities/supervising midwifery students? Which tasks do you delegate to your practice assistant, and are you satisfied with that? c. Do you think that the following tasks should be executed by midwives? Advise in education possibilities, discuss relational or sexual problems, offer help in case of tendency towards suicide, discuss problems at work, discuss lifestyle (eating, drinking, smoking). Do you (dis)agree with statements on job satisfaction, e.g., my job is useful, my job makes me satisfied, I am enthusiastic about my job, my job is interesting, I have enough time to provide good care. With what items of your current job are you happy, and what can be improved? d. How often do you look up information in guidelines of the Royal Dutch Organisation of Midwives (KNOV)? How much time do you spend on training/education for yourself? Are you registered in one of the quality registries of the KNOV? How much time do you reserve for: each intake, preconception consult, regular consult without ultrasound scan, regular consult with ultrasound scan, counselling regarding prenatal screening, postpartum consult? e. How many years of working experience do you have as a midwife? How many hours per week are you currently working? Do you provide preconception care?

Midwifery practices	June 2010	61	a. organisation of the practice b. size c. provision of preconception information d. collaboration and meetings with health care providers within and outside the practice e. placements and education of midwifery students f. care provided to ethnic minority women and undocumented women	a. number of employees/associates, distribution of tasks, time reserved per client visit, computerised activities and databases, presence and tasks of a practice assistant b. annual number of new clients and deliveries c. frequency of preconception consults d. frequency and duration of regular meetings with care providers within and outside your practice hospitals where clients are referred to and distance to hospitals e. annual number of midwifery students, medical students, nursing students f. annual number of ethnic minority women and undocumented women

* Q0: socio-demographic characteristics, asked simultaneously with first questionnaire that a client completes; Q1: 1^st ^questionnaire, before 35 weeks gestation; Q2: 2^nd ^questionnaire, after 35 weeks gestation; Q3: 3^rd ^questionnaire, postpartum

Virtually all midwives in the Netherlands routinely submit data about mothers, newborns, and their care provision to the Netherlands Perinatal Registry [3]. The midwifery practices that participated in the DELIVER study sent these data, of all clients that completed at least one questionnaire, to the DELIVER research team electronically (Figure 1). These data included information on the mother (e.g., age, gravity, parity), birth (e.g., pain management, duration of labour, complications), baby (e.g., time of birth, birth weight, Apgar score at 5 minutes), and provided care (e.g., place of birth, referral to secondary care).

The midwifery practices also sent electronic client records data of participating clients to the research team including demographic information, medical history, progress of pregnancy (e.g., blood pressure of mother, foetal heart rate, position of baby, health status of the mother), and care plans (e.g., place of birth, breast or formula feeding) (Figure 1).

#### Measurement tools administered among clients

Clients received up to three questionnaires depending on their gestation at inclusion: one before 35 weeks gestation (completed on average around 20 weeks gestation), one between 35 weeks gestation and birth, one about 6 weeks postpartum. The primary aim of the questionnaires was to assess the expectations and experiences of clients regarding midwifery care. The questionnaires included validated instruments used in earlier studies (Table 2). The questionnaires were largely based on two National Surveys in general practice, which were conducted by the NIVEL institute [4]. The issues that were covered by the questionnaires as well as examples of items are given in Table 1.

**Table 2:** Validated measurements used in client questionnaires

Measurement	Goal	Content	Client questionnaire*
Bologna score [8]	To determine whether the intrapartum care in case of a normal birth was according to the best evidence.	5 items: - presence of partner or friend during labour - use of a partogram (measure progression objectively) - absence of interventions - labour not in supine position - skin-to-skin contact between mother and child for at least 30 minutes during first hour postpartum	Q3

Dutch consumer quality index (CQI) treatment score [9]	To measure the actual experience of clients with structure and process aspects of health care, as well as the importance clients attach to each aspect.	6 items: - Does your *midwife *treat you with respect? - Do you feel that your *midwife *listens to you? - Does your *midwife *devote enough time to you? - Do you feel that your *midwife *takes you seriously? - Does your *midwife *explain things to you in a way that is easy for you to understand? - Do you feel you are in good hands with your *midwife*? (options: never/sometimes/usually/always)	Q2 + Q3

Labour Agentry Scale [10]	To measure personal control during childbirth (separately during first and second stage of labour).	10 items (shortened version): - I was tense - I felt important - I felt confident - I felt I was in control of myself - I was scared - I was relaxed - I felt I was doing a good job - I felt helpless - I felt powerless - I felt I was surrounded by people who cared for me - I felt a failure (options: The whole time or nearly the whole time/About three quarters of the time/Just over half the time/About half the time/Just under half the time/About a quarter of the time/Not or hardly at all)	Q3

EuroQol questionnaire [11]	To measure health-related quality of life, categorized by mobility, self-care, main activity, social relationships, pain and mood.	6 dimensions: - Mobility - Self-care - Main activity (eg work, study, housework) - Social relationships (pursue family and leisure activities) - Pain - Mood (anxious or depressed)	Q1 + Q2 + Q3

Visual Analogue Scale [12]	To measure pain	10 cm visual analogue scale, from 'no pain' to 'worst pain imaginable'	Q2 + Q3

* Q1: 1^st ^questionnaire, before 35 weeks gestation; Q2: 2^nd ^questionnaire, after 35 weeks gestation; Q3: 3^rd ^questionnaire, postpartum

A pilot study took place from May to July 2009 in three midwifery practices to test the client questionnaires. During the pilot study, 710 clients completed 774 questionnaires. The content of the questionnaires was adjusted according to comments made by clients during the pilot phase (e.g., too long, some questions were experienced as depressing) in August 2009.

As the response rate of clients from ethnic minority groups was relatively low in the pilot study, a lot of effort was put into reaching such women during the main part of the study, especially women with a Turkish or Moroccan ethnic background. Primarily, the questionnaires were only offered on-line in Dutch. To enlarge participation of women from deprived areas and from ethnic minorities, written questionnaires were developed in Dutch and English [13]. As the largest groups of non-Western women in the Netherlands are from Turkish or Moroccan origin, the questionnaires were translated into Turkish and Arabic and the services of an interview bureau were enlisted to conduct telephone interviews in these languages.

Midwives and research assistants tried to collect information about all non-participating clients on age, parity, ethnicity, postal code (to determine socioeconomic position), and reason for non-participation. This information can be used to check the external validity of the data.

Video recordings of intake consults in primary care midwifery practices were used to gain insight into daily practice of primary care midwives, mainly on counselling regarding prenatal screening. The Roter Interaction Analysis System (RIAS) was used, which is a method of coding doctor-patient interaction during the medical visit [14]. In addition, content analysis was used for the following subjects: lifestyle (smoking, alcohol, weight (gain), nutrition), drug use during pregnancy, infectious diseases, and demographic information. Also, global affect ratings were recorded for the consult as a whole to rate the affect or the emotional context of the dialogues.

#### Focus groups with partners

The research team of the DELIVER study developed a topic list which was used to evaluate the expectations, needs and experiences of partners of pregnant women regarding the care provided by midwives. The topic list comprised amongst others midwives' characteristics, counselling regarding prenatal screening, preparation for labour, the midwife's role during labour, and postnatal care.

#### Interviews with other maternity care providers

Telephone interviews were held with health care professionals that work together with primary care midwives, namely clinical midwives, gynaecologists, general practitioners, maternity care assistants, paediatricians, ambulance personnel, and O&G nurses. The main aim of these interviews was to gain insight into the number of contacts, reasons for contact, and their views on the quality of the contacts. The aim was to interview three people per profession.

### Data management

#### Data at midwife or practice level

The midwives and practice assistants sent their completed diaries with work-related activities to the NIVEL institute where the data were (partly) analysed. The data and results were then passed on to the department of Midwifery Science of VU University Medical Center (Figure 1).

The written questionnaires that were completed by the midwives of the twenty participating practices were returned to the department of Midwifery Science. Also the questionnaires that were completed by more than half of all midwifery practices across in the Netherlands were sent to the department of Midwifery Science. Data from these questionnaires were entered into SPSS by a research assistant.

Midwives received no information about study results during the course of the study to avoid bias in midwifery practice, with the exception of client response rates which were provided in order to promote further motivation in encouraging participation among their clients.

#### Data at client level

Client data from written questionnaires and from telephone interviews were entered into the on-line questionnaire by research assistants. The research bureau converted all data from the client questionnaires into an SPSS database, which was transferred to the department of Midwifery Science (Figure 1).

The midwifery practices sent the data of the Netherlands Perinatal Registry and the data of the electronic client records to the NIVEL institute at the end of the study (Figure 1). The NIVEL institute converted these data into an SPSS database before releasing the data to the department of Midwifery Science.

The video tapes were analysed using a special program at the NIVEL institute. The original tapes stayed at NIVEL, but the analysed data were converted into an SPSS database and sent to the department of Midwifery Science.

#### Focus groups with partners & interviews with other maternity care providers

The focus groups with partners and the interviews with other maternity care providers were conducted and analyzed by the research bureau Intomart GfK. They processed the results of their analyses into reports which were sent to the department of Midwifery Science. Original data remained property of Intomart GfK but the anonymous transcriptions of the interviews with partners and other maternity care providers were sent to the department of Midwifery Science.

#### Check data entry

Data that were entered manually (on-line or into SPSS) by someone from the research team or by a research assistant were checked by another person. A randomly selected sample of 5% of the questionnaires and client records was checked for errors and the error rate was below the maximum tolerated error rate of 1% at item level.

#### Data linkage

A crucial aspect of the data collection within the framework of the DELIVER study is the possibility to link the data. The overall database consists of data at three levels: individual client, individual midwife, and midwifery practice. These data were linked by unique anonymous client identifiers and anonymous midwifery practice identifiers.

### Data analyses

#### Power

The number of included midwifery practices (*n *= 20) was based on experience of two National Surveys of General Practice in the Netherlands [4]. A priori, we expected an average of 360 new clients annually per midwifery practice and therefore a total of 7200 women in the twenty participating practices during the course of the study. For about only a quarter of these clients (*n *= 1800) it would be possible to complete all three questionnaires during the one-year study period. We aimed for a response rate of 60%, which was based on experience with the two National Surveys of General Practice.

Furthermore, we expected all midwives in the twenty practices to complete a questionnaire and expected all midwives and their practice assistants to complete a diary of work-related activities. Regarding the questionnaire that was sent to all midwifery practices in the Netherlands, we aimed for a response rate of 50%.

We aimed for at least ten video recordings of intake consults per midwife, because the reliability of the results increase with increasing number of video's per midwife. We decided to include six midwifery practices in order to be able to detect possible differences between practices.

Two focus groups were held with partners of women expecting their first child and two focus groups with partners of women who already had at least one child, because we wanted to have data from both groups separately. Besides, we aimed to do a qualitative survey, in other words, get an overview of themes that are important to partners regarding maternity care. For this purpose, this sample was considered to be sufficient [15,16].

Regarding interviews with other maternity care providers, we interviewed people from a wide range of maternity care providers to get maximum variation in the research sample. It was decided to interview three people per category (21 in total), because we felt that there would be considerable overlap in themes between provider groups, and that saturation would be reached with this number of interviews.

#### Data analyses

The different modes of data collection require different types of data analyses, e.g. qualitative analyses for interviews and focus groups, quantitative analyses for questionnaires and diaries, and content and interaction analyses for video recordings. Subsequent publications reporting the study results will provide plans for the data analyses in detail. These subsequent publications will also report on representativeness of the clients, midwives, and midwifery practices that participated in the DELIVER study. For that purpose, characteristics of the participating midwifery practices will be compared with non-participating practices concerning region, urbanisation, and size of the practice (number of midwives and annual number of clients). In order to determine whether the midwives from the twenty participating practices are comparable with all other Dutch midwives, national data will be obtained from the NIVEL Institute on age, years of experience as a midwife, and weekly working hours. Data from Statistics Netherlands will be used to assess the representativeness of the participating clients regarding age, parity, social-economic status, education, religion, and ethnicity [17]. Because of all made efforts to obtain representative populations in the DELIVER study, we do not expect major selection bias. If comparisons with national populations do reveal selection bias, we will correct for this by adjusting results for relevant confounders. The available background information on clients and midwives will make it possible to adjust results for confounders such as age, parity, ethnicity, education, income, presence of a partner, and religion. The cluster design will be taken into account by applying multilevel modelling when necessary and possible.

As DELIVER is a descriptive study, the sample size can best be based on the width of the confidence interval around estimates. Of all women, 1890 filled in all three questionnaires. Taking clustering of data in twenty midwifery practices into account and assuming an intraclass correlation of 0.015, the effective sample size is 789. The width of the 95% confidence intervals around point estimates for continuous variables will be 0.14 times the standard deviation. For example, if we find a mean of 9 prenatal visits and a standard deviation of 2.9, the 95% confidence interval will be 8.8 to 9.2.

Regarding the first research question (organisation of care), descriptive analyses will be conducted using data from midwives' diaries with work-related activities, the questionnaire they completed, and interviews with other maternity care providers. These data sources reveal information on collaboration and meetings with health care providers within and outside the practice, number of employees/associates, presence of a practice assistant, distribution of tasks, annual number of new clients and deliveries, frequency of preconception consults.

For the second research question (accessibility of care), descriptive analyses will be conducted with the main variables being timing of first consult, accessibility of practice by phone, accessibility of practice by public transport, availability of consults outside office hours, and the provision of care to ethnic minority women and undocumented women.

Regarding the third research question (quality of care), the main variables will be satisfaction of clients and their partners, midwives' adherence to standards and guidelines, quality and content of intake consults, quality of collaboration with other maternity care providers, and pregnancy outcomes.

### Ethical approval and privacy issues

The design and conduct of the study were offered to the Medical Ethics Committee of the VU University Medical Centre Amsterdam. Participating midwifery practices were expected to participate in all aspects of the DELIVER study. Client participation was voluntary and they could withdraw at any time.

Privacy was guaranteed in accordance with Dutch legislation. Clients' and midwives' anonymity was maintained by using anonymous patient and practice identifiers.

### Incentives

We estimated that the time investment for midwives would be about 1.5 hours per week for an average practice. Each participating midwifery practice received on average €2,000 for their input, depending on their annual number of clients. In addition, the practices received several presents during the course of the study to keep them motivated. We also tried to keep the midwives enthusiastic by sending them regular news letters with stories from midwives or researchers, tips to increase the client response rate, clients' response rates per practice, and frequently asked questions.

All clients who complete at least one questionnaire received shower gel. Additionally, five coupons worth of 100 euro were raffled among all clients who completed at least two questionnaires. Client's partners who participated in one of the four focus groups received a gift certificate of 35 or 40 euro's.

## Results

An overview of the collected data within the DELIVER study is given in Table 3. Ultimately, 34 midwifery practices were approached in order to achieve the sample of twenty practices that were willing to participate. The stratification criteria for selection of participating midwifery practices, led to a representative sample of twenty practices regarding region (5 north, 6 east, 3 south, 6 west), practice type (2 dual and 18 group practices), and level of urbanisation (5 urban area, 6 rural area, 9 combination of urban and rural area).

**Table 3:** Collected data

Level	Measure	Subjects	Number of participants (%)
Client	Questionnaires (max 3)	All clients in 20 participating practices (during one year)	7685 (53%*)

	Netherlands Perinatal Registry	All clients that completed at least one questionnaire	5913 (77%)

	Electronic client records	All clients that completed at least one questionnaire	5895 (77%)

	Video recordings	Midwives + clients during first consult	310 clients/23 midwives/6 practices

	Focus groups	Partners of clients	30

Midwife	Questionnaire	All midwives in 20 participating practices	99 (92%)

	Diary of work-related activities (one week)	All midwives + practice assistants in 20 participating practices	136 (100%)

Practice	Questionnaire	All 521 midwifery practices in the Netherlands	319 (61%)

Other	Interviews	Other maternity care providers (clinical midwives, gynaecologists, general practitioners, maternity care assistants, paediatricians, ambulance personnel, and O&G nurses)	21

* If women with an abortion, a miscarriage or intra uterine death were excluded from the denominator, the netto response rate would be estimated to be 62%

Of all 14418 invited clients, 7685 clients participated by returning at least one questionnaire and 1890 clients returned all three questionnaires. Most questionnaires were completed online, but 25% of the completed questionnaires after labour were print questionnaires. The interview bureau interviewed 183 Turkish and Moroccan clients. The overall crude client response rate was 53%. However, in this calculation women with an abortion or miscarriage were included in the denominator while these women were actually not part of our study population. Data from a part of the non-participants (*n *= 922) showed that 30% of them did not want to participate in this study because of an abortion or a miscarriage. If we assume that 30% of all 6733 non-responders (*n *= 2020) would have an abortion or a miscarriage, and therefore were not eligible for our study, the adjusted response rate is 62% (7685/12398). Data of the Netherlands Perinatal Registry could be linked to questionnaires for 5913 women, and the data of the electronic client records for 5895 women, and both registries for 5133 women.

For each specific research question, different client data might be included. Therefore, the representativeness of the client population will be considered for each research question separately. Overall, the distribution of participating clients over the country (26% north, 30% east, 15% south, 30% west) was comparable with the distribution of the national population in the Netherlands. Seventeen percent of the DELIVER client population was of non-Dutch origin, compared with 25% of the national female population between 15 and 45 years of age in 2010 (*p *< 0.05). More specifically, the DELIVER client population comprised 4.3% Turkish and Moroccan clients, compared to 5.9% nationally (*p *< 0.05).

Regarding the questionnaire for midwives in the twenty practices, 99 of the 108 midwives completed the questionnaire (92%). All 108 midwives and 28 assistants in the twenty practices completed a diary. Regarding the questionnaire that was sent to all 521 Dutch midwifery practices, 319 practices returned the completed questionnaire (61% response rate).

In six midwifery practices, 310 video recordings were made of intake consults. This concerned in total 23 different midwives.

Thirty partners of pregnant women participated in one of the four focus group interviews. Twenty-one health care professionals that work together with primary care midwives were interviewed, namely four general practitioners, two ambulance personnel, and three of each of the remaining five professions (i.e. clinical midwives, gynaecologists, maternity care assistants, paediatricians, and O&G nurses).

## Discussion

The DELIVER study is the first study evaluating the quality and provision of primary care midwifery in the Netherlands on such a large scale. The Dutch maternity care system is rather unique with a high number of homebirths and primary midwifery led births. In many countries midwives look at the Dutch system for inspiration. It is therefore crucial that the quality and characteristics of this system are described and that this information is put out in the public arena to inform people internationally about its advantages and disadvantages.

The DELIVER study protocol is presented in the present paper to offer researchers the opportunity to critically review the methodological quality of this study. A discussion of the methodological issues of the DELIVER study follows below.

Research into primary care midwifery can make an important contribution to the improvement of prenatal, intrapartum, and postnatal care by midwives and thus contribute to the safety and satisfaction in childbirth. Midwives are considered to be important care providers of pregnant women in the Netherlands as 78% of clients start prenatal care at the primary care level and pregnant women have frequent contacts with midwives throughout pregnancy and after childbirth [3]. The DELIVER study provides evidence about the strengths and weaknesses of the current maternity care system regarding the quality, organisation and accessibility of primary care midwifery, which gives insight into areas for improvement that might lead to improved safety and satisfaction in childbirth. In addition, results of the DELIVER study should enhance evidence-based practice and may contribute to the start of a new continuous registration system in midwifery practices in the Netherlands. Such a continuous registration system will provide easy-accessible data for structural research on various aspects concerning primary care midwifery. Furthermore, data collection forms and experiences of the DELIVER study are currently used to establish a client panel of 1000 pregnant women in order to regularly collect data on their experiences with care and their health and well being.

By including exhaustive information from 7685 pregnant women and 108 midwives from 20 midwifery practices plus data from 299 other primary care midwifery practices, the DELIVER study has led to a rich and substantial dataset which will allow description of various aspects of maternity care from the perspectives of midwives as well as their clients, clients' partners, and other relevant maternity care providers, making it a multidisciplinary study.

Clients from ethnic minority groups were underrepresented in the pilot study, mainly because the questionnaire was only available in Dutch at that time. In the main study, many protocol adjustments and additional actions were executed to increase the response rate of this specific population: printed questionnaires were developed in Dutch and English and services of an interview bureau were enlisted to conduct telephone interviews in Turkish and Arabic. The inclusion of Dutch-speaking clients as well as non-Dutch speaking clients who could understand English, Turkish or Arabic in the main study, increased the external validity of the results because it enabled the four largest non-Western minority groups in the Netherlands to participate (women from Moroccan, Turkish, Surinamese and Antillean origin) as well as many other minority women who speak English.

The use of on-line questionnaires, which were used for the client questionnaires, was very advantageous because built-in checks and a logical follow-up of questions led to a low rate of missing data or errors (e.g., women could only give one answer and within the pre-set range of possible answers), and data could easily be uploaded to SPSS. However, in order to improve response rates for the client questionnaires, the clients were offered a choice between electronic and print questionnaires in either Dutch or English. The fact that many clients used this opportunity (25% of the completed questionnaires after labour were print questionnaires) indicates that this was a useful option to include.

The video recordings that we made of 310 client consults by 23 midwives in six practices provide unique data, and this method of data collection has hardly ever been used before in midwifery care research. It is probably the optimal way to objectively evaluate the daily practice of midwives.

Because not all data have been analysed yet, we cannot currently give insight in the strengths and weaknesses of the maternity care system in the Netherlands regarding the quality, organisation and accessibility of primary care midwifery. In process of time, results of the DELIVER study will describe the current level of service, which is the first step to improve midwifery care. The DELIVER study provides unique data on the activities of midwives, the variation between them and the evaluation of their care by clients and other maternity care providers. These data will enhance awareness among midwives about the care they give and this in itself may change clinical practice. For example, the results will show how many ultrasound scans women have on average and how this number varies between midwifery practices. If the variation is large, this will likely initiate a debate on when ultrasound is indicated. Secondly, the data from the client questionnaires will provide information on areas of care that could be improved. The Dutch Organisation of Midwives might use the results when developing their practice guidelines. Thirdly, if changes are introduced in midwifery practice, a repetition of the DELIVER study can show the extent to which these changes have materialised by making a comparison between the results of the first and second DELIVER study.

Certainly, the DELIVER study will enhance evidence-based practice in primary midwifery care in the Netherlands. And regarding the current global discussion about the organisation of maternity care (e.g. place of birth), this study provides a reliable basis for future research.

## Conclusion

The multicenter multidisciplinary DELIVER study provides an extensive database with nationally representative data on the quality of primary care midwifery in the Netherlands. This study will support evidence-based practice in primary care midwifery in the Netherlands and will contribute to a better understanding of the maternity care system and provide scientific knowledge for improvement.

## Competing interests

The authors declare that they have no competing interests.

## Authors' contributions

ES, FS, and MM originated the idea for the study. ES and TK supervised the study. TK and MP recruited the midwifery practices. TW was responsible for many measurement instruments (including diaries with midwives' work-related activities) and data linkage. JM monitored the data collection. All authors participated in discussing the design of the study and developing the research protocols and questionnaires. The core research team consisted of ES, TK, FS, TW, MP, and JM. JM drafted the manuscript, and all authors read and corrected draft versions of the manuscript and approved the final manuscript.

## Query

1. Abstract: Journal standard instruction for structure Abstract requires the sub-section "Background; Methods/Design; Discussion" for article type "Study protocol". Hence, "Methods" was change to "Methods/Design" sub-section heading. Please check if action taken is appropriate. Otherwise, kindly advise us on how to proceed.

2. Article structure: Journal standard instruction regarding article structure requires the section "Background; Methods/Design; Discussion" for article type "Study protocol". Hence, "Methods" was change to "Methods/Design" section heading. Please check if action taken is appropriate. Otherwise, kindly advise us on how to proceed.

3. Additional files: Provided additional files 1, 2 and 3 contain Tables. On the other hand, Tables 1, 2 and 4 were mentioned in the manuscript; however, no e-files were received. Hence, the additional files were captured as normal Table 1, Table 2 and Table 3. Please verify if action taken is appropriate. Otherwise, kindly advise us on how to proceed.

4. References: In reference entry [4], the author name was provided as "van der ZJ". This was changed to "van der Zee J". Please check and advise if action taken is appropriate.

## Pre-publication history

The pre-publication history for this paper can be accessed here:

http://www.biomedcentral.com/1472-6963/12/69/prepub
